# Quantitative Assessment of Head Motion toward Functional Magnetic Resonance Imaging during Stepping

**DOI:** 10.2463/mrms.mp.2015-0015

**Published:** 2015-11-06

**Authors:** Kousaku SAOTOME, Akira MATSUSHITA, Kei NAKAI, Hideki KADONE, Hideo TSURUSHIMA, Yoshiyuki SANKAI, Akira MATSUMURA

**Affiliations:** 1 Center for Cybernics Research, University of Tsukuba 1-1-1 Tennoudai, Tsukuba, Ibaraki 305-8577, Japan; 2 Graduate School of Comprehensive Human Science Majors of Medical Sciences, University of Tsukuba; 3 Department of Neurosurgery, University of Tsukuba; 4 Graduate School of Systems and Information Engineering, University of Tsukuba

**Keywords:** head movements, motion, kinesis, magnetic resonance imaging, quantitative assessment

## Abstract

**Purpose:**

Stepping motions have been often used as gait-like patterns in functional magnetic resonance imaging (fMRI) to understand gait control. However, it is still very difficult to stabilize the task-related head motion. Our main purpose is to provide characteristics of the task-related head motion during stepping to develop robust restraints toward fMRI.

**Methods:**

Multidirectional head and knee position during stepping were acquired using a motion capture system outside MRI room in 13 healthy participants. Six phases in a stepping motion were defined by reference to the left knee angles and the mean of superior-inferior head velocity (*V_mean_*) in each phase was investigated. Furthermore, the correlation between the standard deviation of the knee angle (*θ_sd_*) and the maximum of the head velocity (*V_max_*) was evaluated.

**Results:**

The standard deviation of each superior-inferior head position and pitch were significantly larger than the other measurements. *V_mean_* showed a characteristic repeating pattern associated with the knee angle. Additionally, there were significant correlations between *θ_sd_* and *V_max_*.

**Conclusions:**

This is the first report to reveal the characteristics of the task-related head motion during stepping. Our findings are an essential step in the development of robust restraint toward fMRI during stepping task.

## Introduction

Stepping motion has often been used as the multi-joint leg task in functional magnetic resonance imaging (fMRI).^1–3^ This consists of coordinated movements where both legs extend and flex alternately and comprise multi-joint interlocking movements of the hip, knee, and ankle joints. Complex motion activates wide regions of the primary motor cortex, premotor cortex, supplementary motor cortex, and sensorimotor cortex.^4–9^ These studies are important as they challenge our understanding of gait control in healthy participants and patients with gait disorders.

fMRI using blood oxygenation level dependence is a common approach to the imaging of regions involved in cognition and motor control, and is now widely used throughout neuroscience.^10–13^ It has advantages over positron emission tomography, single photon emission computed tomography, and near-infrared spectroscopy in that it does not require the administration of a contrast medium, and acquires high-resolution images. In fMRI, the translational and rotational head motion during image acquisition is a major source of motion artifact and makes it very difficult to assess brain activity.^14–17^ Past studies have attempted to suppress head motion using restraints, however, these are still challenging.^1,2,18–20^ Moreover, a number of strategies using fast acquisition have been developed in recent years.^14,15,21–25^ However, these techniques cannot often acquire satisfactory images because of excessive head motion.

To develop the robust restraint for stepping motion toward fMRI, the investigation of the characteristics of head motion during stepping is required. Seto et al. quantitatively showed the amount of head motion during hand and ankle tasks in fMRI in detail.^26^ Though head motion tends to increase during multi-joint movement tasks such as stepping than single joint tasks,^1,2,26–29^ the quantitative assessment of head motion during stepping has not been investigated. The development of the task-suitable restraint for stepping could be an essential step towards the research of brain function for gait control using fMRI.

Our study measured the head and leg motion (three orthogonal translation directions and three rotations) of healthy participants during stepping, and investigated the relationship between the head position and the knee angle, velocity, using a motion capture system. Measurements were performed in a motion capture laboratory outside the MRI roomand used 12 cameras to acquire multidirectional head position and the knee angle data. Our data provide accurate and detailed three-dimensional information for the head positions and knee angles, and reveal the characteristics of head motion.

## Materials and Methods

### Subjects

Thirteen young healthy male volunteers participated in this study. The mean ± standard deviation of the participants’ age was 23.2 ± 2.5 years. Each subject gave written informed consent before entering this study. The protocol was approved by the University of Tsukuba Ethics Committee (No. 745). Prior to participating, volunteers were also screened using checklists. Participants were excluded if they had a history of neurological impairments or physical conditions contraindicative to exercise.

### Experimental setup

#### The couch:

**Fig. 1.**
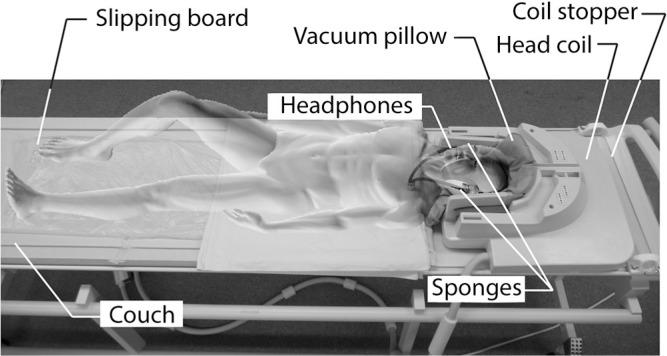
Couch setup. The 32-channel SENSE head coil was set on the couch. The anterior part of the head coil was displaced to measure the head position by motion capture. A homemade coil stopper was placed above the head coil to prevent it from sliding. A slippery board made of acrylic and wrapped in a polyethylene bag was aligned on the bed so that it would touch the soles during stepping to achieve fluid motion of the legs. The participant was positioned on the scanner bed in a supine position and their head was placed in the head coil. The head was restrained using sponges, a beaded vacuum pillow, and magnetic resonance-compatible headphones, which are generally used in most magnetic resonance imaging examinations.

The couch, which was the same shape as the one in the MRI scanner (Philips Medical Systems, Eindhoven, The Netherlands), was set up in a motion capture laboratory outside the MRI room (Fig. 1). A 32-channel SENSE head coil was set on the couch, but the anterior part of the head coil was displaced to allow measurement of head position by motion capture. A homemade coil stopper was placed at the top of the head coil to prevent the head coil from sliding. Participants wore socks. A slippery board made of acrylic and wrapped in a polyethylene bag was aligned on the bed so that it would touch the soles during stepping to allow fluid motion of the legs. Participants were positioned on the scanner bed in a supine position and their head was placed in the head coil. The head was restrained using sponges and a beaded vacuum pillow (Tatsuno Cork Industries Co. Ltd., Hyogo, Japan) formed to the shape of each participant’s head, and magnetic resonance compatible headphones, which are generally used in most MRI examinations.

#### The motion capture system:

**Fig. 2.**
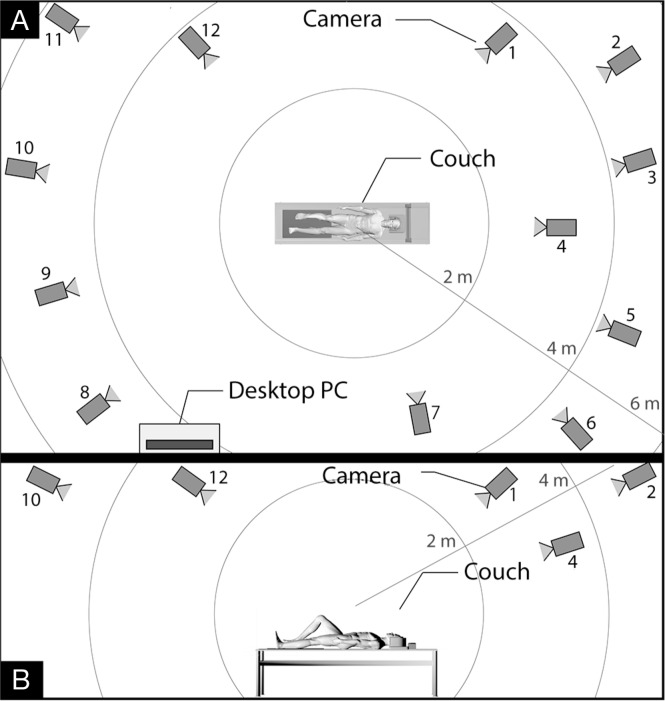
(**A**) Top view and (**B**) side view of the motion capture laboratory. The 12 cameras were set around the couch. The desktop computer with operating software was set unobtrusively on the edge of the examination room.

**Fig. 3.**
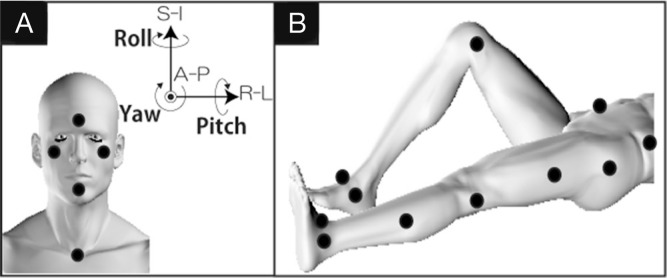
Optical marker positions on (**A**) the face and (**B**) the legs. On the face, markers were placed on the middle of the forehead, the right and left cheekbones, and the chin. On the legs, markers were placed bilaterally on the greater trochanter and lateral epicondyle of the femur and the lateral malleolus of the fibula. Additionally, a marker that was used for supportive purposes in the three-dimensional reconstruction of the other makers was placed on the middle of the clavicles, and five additional markers were also placed on the iliac crest, thigh, medial epicondyle of the femur, shank, medial malleolus, and instep of both legs. Each three-rotation (roll, pitch, yaw) and three-translation (S-I; superior-inferior, R-L; right-left, and A-P; anterior-posterior) was defined as shown.

The motion of the head and the lower limbs was measured using an MAC3D motion capture system (Motion Analysis, Santa Rosa, CA, USA) in a laboratory outside the MRI room. The system consisted of 12 Raptor-4 (2352 × 1728 pixels) cameras arranged around the couch, and a desktop computer with operating software (Fig. 2). Optical markers were placed on the face of the participant on the middle of the forehead, the right and left cheek bones, and the chin, to compute each three-rotation (roll, pitch, yaw) and three-translation [S-I; superior-inferior, R-L; right-left, and A-P; anterior-posterior of the head (Fig. 3A)]. Markers were also placed bilaterally on the greater trochanter, the lateral epicondyle of the femur, and the lateral malleolus of the fibula to obtain the flexion/extension angle of the knee joint (Fig. 3B). Additionally, a marker was placed on the middle of the clavicles, and six additional markers were also placed on the iliac crest, thigh, medial epicondyle of the femur, shank, medial malleolus, and instep of both legs (Fig. 3B). These were used for supportive purposes in three-dimensional reconstruction of the other markers. The system was calibrated following a standard procedure guided by the software provided by the supplier. After calibration of the camera position and orientation, the residual error in the reconstruction of the three markers on the wand, which was used to collect data for calibration, was 0.539 mm on average with 0.224 mm standard deviation throughout the whole capture area. The system recorded the marker positions at 120 Hz (approximately 8 ms/fr), and the data was calculated in each 56 ms. This is because 56 ms is realistic situation of one slice acquisition for time resolution in typical fMRI sequence.

### Stepping task

**Fig. 4.**
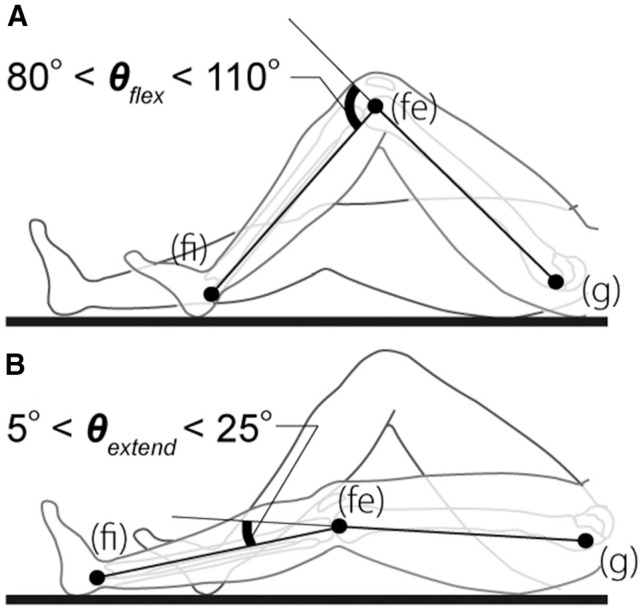
(**A**) Flexion target angle (*θ_flex_*) and (**B**) extension target angle (*θ_extend_*) of the knee joints. *θ* is defined as the angle between the line connecting the greater trochanter of the femur with the lateral epicondyle of the femur and the line connecting the lateral epicondyle of the femur with the lateral epicondyle of the fibula. (g), the greater trochanter of the femur; (fe), the lateral epicondyle of the femur; (fi), the lateral epicondyle of the fibula

All participants performed stepping in supine position. Participants were instructed as follows: (1) the start position was to entirely extend both legs; (2) the left leg was first flexed; (3) when the knee joint angle (θ) was defined as the angle between the line connecting the greater trochanter of the femur with the lateral epicondyle of the femur and the line connecting the lateral epicondyle of the femur with the lateral epicondyle of the fibula, the flexion target angle of the knee joints (*θ_flex_*) was 80° < *θ_flex_* < 110° (Fig. 4A); (4) when one side of the leg started to extend, the other side started to flex; (5) the extension target angle of the knee joints (*θ_extend_*) was 5° < *θ_extend_* < 25° (Fig. 4B); (6) this exercise was continued for 30 s after the cue; (7) stepping was performed at approximately 1.67 Hz (100 beats per second), which was given using a metronome; and (8) participants kept their eyes open to concentrate their gaze on a single point on the ceiling during measurement. Before each measurement, participants practiced the above exercise for 30 s. Moreover, participants were instructed to keep their heads as still as possible during the exercise.

### Data analyses

First, the position of the center of the four markers on the face (middle forehead, right and left cheekbones, and chin), was calculated and used to define the head position. Using these markers’ position, the roll, pitch, and yaw rotation angles of the head was computed, around the S-I, A-P, and P-L axes, respectively (Fig. 3A).

Second, the angles of the knee joints were calculated using the position of the three markers on the legs (greater trochanter of the femur, lateral knee joints, and ankles).

**Fig. 5.**
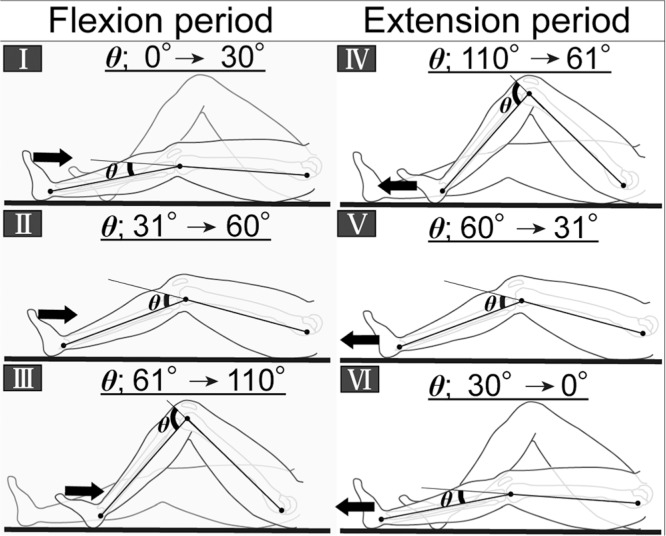
Six phases by reference to the angle of the left knee joint. θ shows the angle of the left knee joint. The flexion period consists of phases I, II, and III. The extension period consists of phases IV, V, and VI; (I) 0° < *θ* < 30°, indicates the beginning of flexion; (II) 31° < *θ* < 60°; (III) 61° < *θ* < 110°, indicates the end of flexion; (IV) 110° < *θ* < 61°, indicates the beginning of extension; and (V) 60°< *θ* < 31°; (VI) 30°< *θ* < 0°, indicates the end of extension.

The below five metrics were used to calculate the head motion during stepping: (1) the standard deviation of the head position (*M_sd_*); (2) the mean of the head velocity (*V_mean_*); (3) the maximum of the head velocity (*V_max_*); and (4) the standard deviation of the angle of the knee joints (*θ_sd_*). *M_sd_* is described by the formula

$$M_{\mathrm{sd}}=\sqrt{\frac{\sum_{i=1}^{N}{\left(X_{i}-\bar{X}\right)}^{2}}{N-1}}$$

where *X_i_* is the head position measurement at a particular time *i*, *X* is the mean of the head position measurements in each of the three orthogonal translation directions or rotations, and *N* is the number of data points. *V_mean_* was calculated as

$$v=\frac{\mathrm{dX}}{\mathrm{dt}}$$

$$V_{\mathrm{mean}}=\frac{\sum_{i-1}^{N-1}v_{i}}{N-1}$$

where *v* is the velocity of the head and *t* is each measurement time. *V_max_* was calculated as

$$V_{\max}={\left|v\right|}_{\max}$$

where *v_max_* is the maximum head velocity in each of the three orthogonal translation directions (S-I; superior-inferior, R-L; right-left, and A-P; anterior-posterior). *θ_sd_* was calculated as

$$\theta_{\mathrm{sd}}=\sqrt{\frac{\sum_{i=1}^{N}{\left(Y_{i}-\bar{Y}\right)}^{2}}{N-1}}$$

where *Y_i_* is the angle of the knee joint at a particular time *i*, and *Ȳ* is the mean of all the knee joint angles. *M_sd_* and *V_mean_* were applied to each of the three orthogonal translation directions (S-I, R-L, A-P) and each of the three rotations (roll, pitch, yaw). Additionally, six phases in stepping were defined by reference to the left knee joint angles to interpret the relation between the head velocity and the angle of the knee joint (Fig. 5); (I) 0° < *θ* < 30°, which indicates the beginning of flexion; (II) 31° < *θ* < 60°; (III) 61° < *θ* < 110°, which indicates the end of flexion; (IV) 110° < *θ* < 61°, which indicates the beginning of extension; (V) 60° < *θ* < 31°; (VI) 30° < *θ* < 0°, which indicates the end of extension. Then, the *V_mean_* in each of the six phases was calculated.

Statistical analyses were performed using SPSS (IBM Statistical Package for the Social Sciences, version 21.0, Chicago, IL, USA). A Mann–Whitney test of three orthogonal translation directions (S-I, R-L, A-P) and three rotations (roll, pitch, yaw) were performed for *M_sd_*. A Mann–Whitney test of the six phases of the knee joint angle was also performed for *V_mean_*. Furthermore, we used the Spearman’s correlation coefficient (r) to evaluate the correlations between *θ_sd_* and *V_max_*. The level of statistical significance for all measures was set at *P* < 0.05.

## Results

### The standard deviation of the head position (M_sd_)

**Fig. 6.**
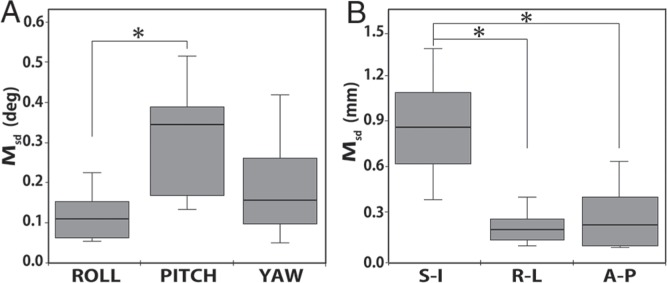
Box-and-whisker plot of the standard deviation of the head position (*M_sd_*) with (**A**) three rotations (roll, pitch, yaw) and (**B**) three orthogonal translation directions (S-I; superior-inferior, R-L; right-left, and A-P; anterior- posterior). Box-and-whisker plots show the median and interquartile ranges of the *M_sd_.* (**A**) There was strong statistically significant difference between roll and pitch. (**B**) There were also strong statistically significant differences between S-I and the others, respectively. Note the difference in vertical scales. *P < 0.001, Mann–Whitney tests.

Figure 6, B are box-and-whisker plots showing the median and interquartile ranges of the standard deviation of the head position (*M_sd_*) with three rotations (roll, pitch, yaw) and three orthogonal translation directions (S-I, R-L, A-P). Among the three rotations, there was a strong statistically significant difference between roll and pitch (*P* < 0.001), but no significant difference between yaw and the others. Furthermore, among the three orthogonal translation directions, there were also strong statistically significant differences between S-I and the others (*P* < 0.001), respectively, but no significant difference between R-L and A-P.

### The relationship between the mean of the head velocity (V_mean_) and the phases of knee angle (I–VI)

**Fig. 7.**
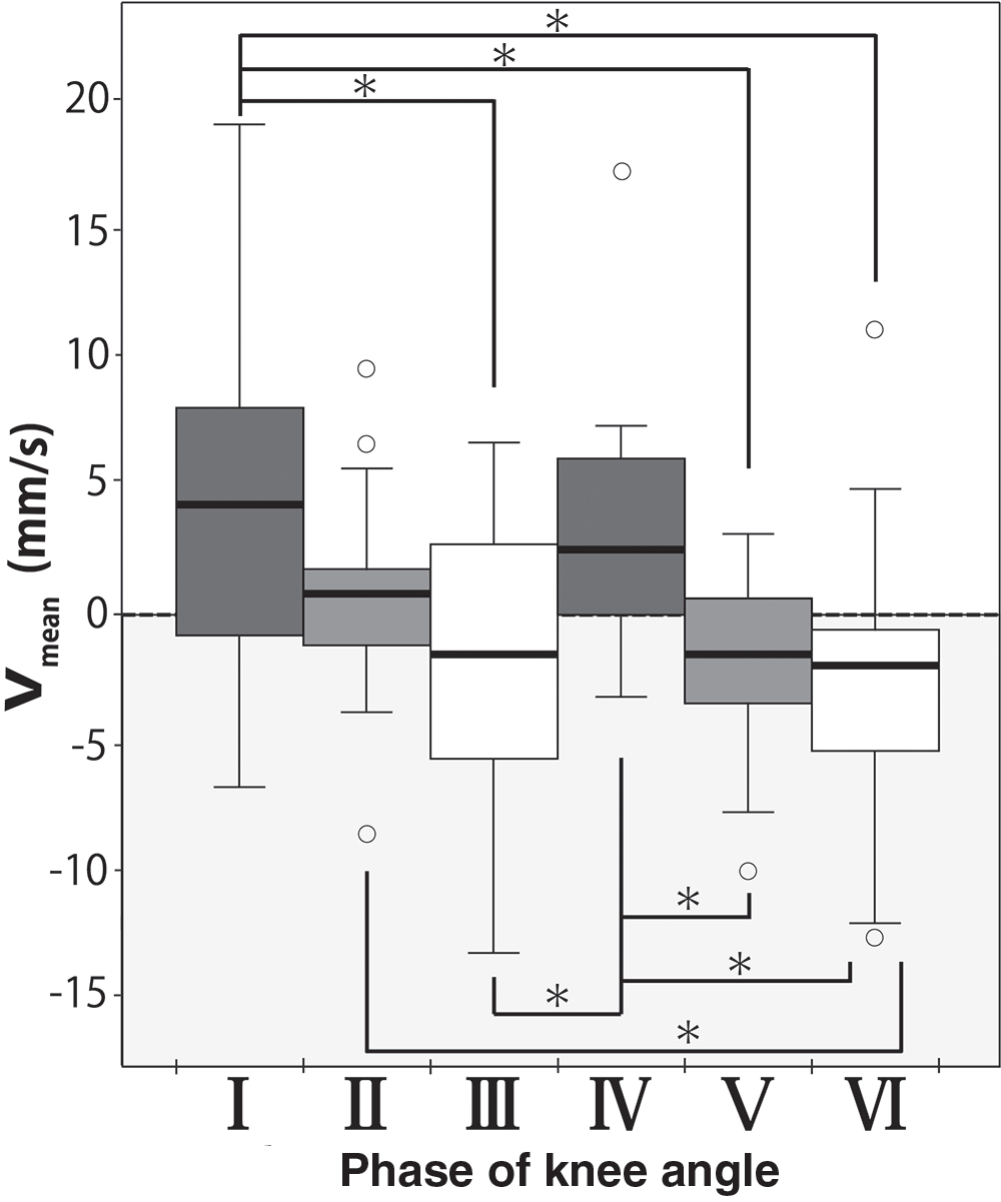
Box-and-whisker plot of the mean of the superior-inferior head velocity (*V_mean_*). Box-and-whisker plots show the median and interquartile ranges of the *V_mean_* in each phase (I–VI) of the left knee angle. There were statistically significant differences between phase I and phases III, V, and VI. There were also statistically significant differences between phase II and phase VI. There were also statistically significant differences between phase IV and phases III, V, and VI. *P < 0.01, Mann–Whitney tests.

Figure 7 is box-and-whisker plots showing the median and interquartile ranges of the mean of the head velocity (*V_mean_*) with S-I in each phase (I–VI) of knee angle. There were statistically significant (*P* < 0.01) differences between phase I and phases III, V, and VI. There were also statistically significant (*P* < 0.01) differences between phase II and phase VI. There were also statistically significant (*P* < 0.01) differences between phase IV and phases III, V, and VI. *V_mean_* of phase I and phase IV showed a larger positive velocity. However, *V_mean_* of phase III and phase VI showed a larger negative velocity. Thus, the larger positive velocity occurred at the beginning of flexion and the beginning of extension. In contrast, these also showed that a larger negative velocity occurred at the end of flexion and the end of extension.

### The correlation between the standard deviation of knee angle (θ_sd_) and the maximum of the head velocity (V_max_)

**Fig. 8.**
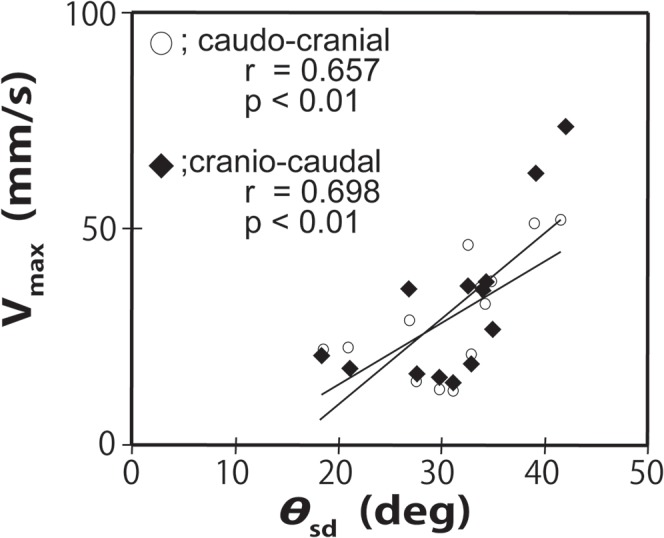
Correlation scatter plot of the standard deviation of left knee angle (*θ_sd_*) in the maximum of the head velocity (*V_max_*). Circles show *θ_sd_* in the caudo-cranial and diamond shapes show *θsd* in the cranio-caudal directions. There were significant correlations between *θ_sd_* and *V_max_* for both caudo-cranial and cranio-caudal directions (r = 0.657, *P* < 0.01 and r = 0.698, *P* < 0.01).

Figure 8 shows the correlations between the standard deviation of knee joint angle (*θ_sd_*) and the maximum of the head velocity *V_max_*. There were significant correlations between *θ_sd_* and *V_max_* for both caudo-cranial and cranio-caudal directions (r = 0.657, *P* < 0.01 and r = 0.698, *P* < 0.01). The mean ± standard deviations of *V_max_* in the caudo-cranial and cranio-caudal directions were 34.1± 14.0 mm/s and 35.4 ± 19.1 mm/s, respectively.

## Discussion

The measurements were performed in a motion capture laboratory outside the MRI room. Prior to an in-depth discussion of our results, we need to describe the differences between the motion capture laboratory and the actual environment within an MRI scanner. The only notable difference was that there were no loud sounds emanating from an MRI scanner in the motion capture laboratory. However, the head motion induced by loud sounds is significantly less than the head motion associated with multi-joint leg movements.^30^ Regarding other differences, there was little influence on the measurements for the following reasons. In the motion capture laboratory, a couch of the same shape and material as that of an MRI scanner was used. In addition, the same head coil, coil stopper, and restraints as those of an MRI scanner were used. Therefore, similar head motion and stepping motion might be possible within the confines of an MRI scanner.

Head motion is dependent on the task and the subject group in fMRI.^1,26^ Seto et al., who investigated the amount of head motion during hand and foot task in stroke subjects and age-matched controls in fMRI, revealed that the head motion in stroke subject with foot task was largest, especially S-I translation. Our result in S-I translation exhibited approximately twice the head motion compared to that of stroke subjects with foot task. Furthermore, all rotation and R-L and A-P translation exhibited the head motion at the same level as that of stroke subjects. Our findings mean that we should pay special attention to suppress the head motion in S-I translation.

Figure 8 shows *V_max_* during stepping in each 60 ms. If the head velocity is 40.0 mm/s, the head moves 2.4 mm during acquiring an echo in a plane, which is a very short period (approximately 60 ms following generally fMRI echo planar imaging sequences; repetition time (TR)/echo time/image matrix/number of slice/slice thickness = 3000 ms/35 ms/128/40 slices/4 mm). This displacement corresponds to 60% of slice thickness. This means that approximately 60% of the plane excited by the slice selection gradient has deviated from its original position at the echo acquisition time on the sequence. Previous studies, which approached fMRI during multi-joint movements of the legs by using trunk restraints in addition to head restraints, stated that the offset value of motion correction algorithm of the SPM software (Wellcome Department of Cognitive Neurology, London, UK) implemented in the Matlab (MathWorks, Natick, MA, USA) has to be limited to a range of 2 mm over a period of a TR to justify the effect of their restraints.^1,2,20,27^ It is reasonable to refer to this value on the SPM software in the single joint movement, however, it would be enough to refer to only this value in multi-joint movement of the legs. This is because that the problems in huge head motion are not only the misregistration of image voxel locations with brain anatomy but also signal loss in a slice plane leading either to false-positive activation or to false-negative activation.^14,15,31^ The motion correction algorithm in the SPM can correct the volume-by-volume displacement acquired in each TR up to a few millimeters. However, this algorithm cannot correct signal loss occurring during acquiring the echoes in a plane. There are many other motion correction algorithms for slice-by-slice and volume-by-volume, however, none of them can correct signal loss in a plane.^21,22,32–35^ Therefore, we should focus on the robust restraint toward fMRI during stepping, which can reduce the head velocity in S-I translation.

The robust restraint against multi-joint movements of the legs is still challenging and should be suiting the characteristics of the task. At the same time, it should be noted that applying excessive pressure on the head could cause severe head pain to the subjects. Some past studies suffered from the head motion associated with the tasks even though they paid close attention to head motion by restraining the head and the trunk.^1,2^ Considering this as well as the problem of causing head pain, some methods to exempt or isolate the force reached into the trunk and the head from the legs might be expected and is the topic of our further study.

Furthermore, our results showed that the head moves repeatedly up and down with regularity in association with the knee angle. This finding is supported by Fig. 7. Mostly positive velocities (caudo-cranial) occurred at the beginning of right extension (phase I) and at the beginning of left extension (IV), and mostly negative velocities (cranio-caudal) occurred at the end of left extension (III) and at the end of right extension (VI), and phases with smaller velocities (II and V) occurred between the phases associated with the positive velocities and negative velocities. These mean that the head moved up and down twice in a stepping cycle, which includes an extension and a flexion for each leg. Here we make an observation that the initial extension of the knee and hip of motion of one leg pushed the head upward, and then the head stopped a while, and then the stretching motion of one leg at the end of extension pulled the head down to its original position. In concurrence with one leg started to flex, the other leg just started to extend and then the head moved up and down once again. Noteworthy, it is interesting that there are two phases including smaller head motion in a stepping. By acquiring image data only in these phases using moderated fast acquisition techniques, the data set with minimum motion might be accomplished. In addition, our findings showed that the larger the knee joint motion range, the larger the head velocity in both caudo-cranial and cranio-caudal. It is therefore important that we need to select an adequate task that has the motion range of knee joint as small as the study’s objective permits.

Understanding the characteristic of head motion during stepping is essential to build the task-suitable restraints in fMRI. To the best of the authors’ knowledge, this is the first report that has quantitatively assessed head motion during stepping in depth. Our future works are the building of the robust restraint and task setting toward fMRI. Then, it could be an essential step toward the investigation of brain function for gait control using fMRI. The limitations of this study are that the measurements were performed outside of an MRI examination room. However, as already described, we assume that similar head motion and stepping motion are possible within the confines of an MRI scanner. Furthermore, only a stepping motion with a single repetition rate as a multi-joint leg movement was performed in this study. Other multi-joint movements and a slower or faster repetition rate might show different results. These cases require further study.

## Conclusion

In this study, the head position and the knee angle during stepping toward fMRI were measured using a motion capture system. All measurements were performed in a motion capture laboratory outside the MRI room to acquire multidirectional head position and knee angle data using a number of cameras. Our results showed the relationship between the head displacement, velocity, and knee angle. During stepping, the superior-inferior translation and pitch rotation were the largest. The mean of the superior-inferior head velocity showed a characteristic repeating pattern associated with the knee angle. There were positive significant correlations between the standard deviation of the knee joint angle and the superior- inferior maximum head velocity. This is the first report that quantitatively assessed the head motion during stepping for fMRI. Our findings might help the building of the robust restraint and the adequate environment against stepping motion to assess brain activity in fMRI.
